# Synthesis and Characterization of Azo Compounds and Study of the Effect of Substituents on Their Liquid Crystalline Behavior

**DOI:** 10.3390/molecules15085620

**Published:** 2010-08-13

**Authors:** Uhood J. Al-Hamdani, Tarik E. Gassim, Howraa H. Radhy

**Affiliations:** Department of Chemistry, College of Education, Basrah University, Basrah, Iraq

**Keywords:** azo, lateral group, liquid crystal

## Abstract

In this paper we present six select mesomorphic azo compounds distinguished by the presence of diverse substituents on a central benzene nucleus. All the synthesized compounds exhibit enantiotropic mesophases. The mesomorphic properties of the substituted compounds were compared with those of the unsubstituted parent compound to evaluate the effect of the nature and position of the substituents on mesomorphism.

## Introduction

Until 1983 it was generally accepted that substituents diminish the mesogenic properties of a compound, with the extent of the effect depending on their size. Cox *et al*. [1] synthesized nematogens in which a phenyl side group was attached without a spacer. Mesogens with aromatic substituents have been prepared [2,3,4,5,6,7]. Recently a homologous series of mesogenic azo compounds containing three rings in the main core and substituted with aromatic or hydroxyl groups on the central benzene nucleus was reported [8,9]. The mesogenic homologous series with hydroxyl group substituents exhibited nematic mesomorphism, whereas the homologous series with aromatic branches exhibited smectogenic tendencies. In the present work, we have introduced a series of OH, Cl, F and CH_3_ substituents into azo compounds in order to study their effect on the mesomorphic properties and transition temperatures. The resulting chemical structures are shown in Figure 1. 

**Figure 1:**
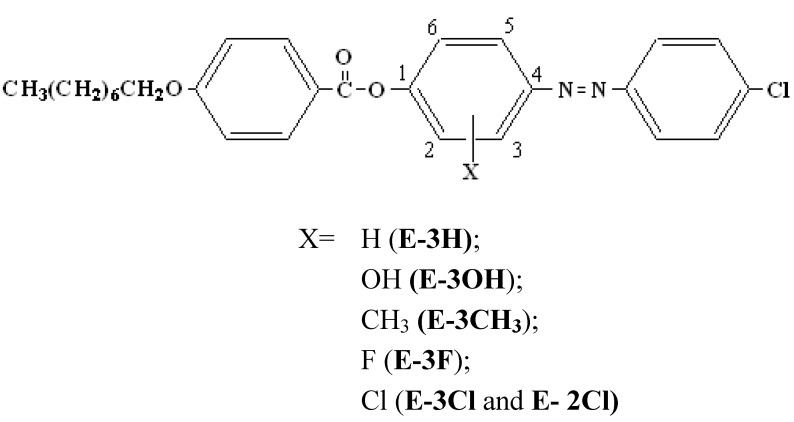
Structures of the studied compounds.

## Results and Discussion

The introduction of a substituent into a side position in the molecule of a mesomorphic compound has two opposing effects on the thermal stability of the mesophase. Firstly, the substituent changes the molecular polarizability and this may increase the thermal stability of the mesophase. However, the size of the side group broadens the molecule and in doing so decreases the length/breadth ratio and hence the mesophase thermal stability. The second of these effects predominates normally, but the destabilizing effect on the mesophase can be minimized by using a small substituent such as fluorine. 

The probability that a compound will exhibit a smectic or nematic mesophase depends on the degree of difference between the terminal and lateral attractions. Smectic properties are most often observed when the lateral to terminal ratio is high and the nematic phase appears when the opposite occurs [10]. The replacement of the hydrogen at the 2– and 3– positions of azo compounds by Cl, CH_3_ and F introduces an additional dipole moment across the long axis of the molecules which enhances the intermolecular lateral attractions so that the smectic phase should appear for the substituted compounds, but instead the observed mesophase is nematic. 

There are possible three reasons for the absence of smectic properties:

(1) The dipole of the substituent X is partly cancelled by the dipole of the ester group which is most likely to lie with its C = O group *trans* to the X group.
(2) There is no other dipole acting across the long axis of the molecule.
(3) As a result of the increase in molecular breadth the long narrow molecules will be forced further apart, thus reducing the strength of the intermolecular lateral attractions***.***

In the case of the compound **E-2Cl**, the chloro atom is attached to an aromatic ring. The effect of the chlorine atom (or for that matter any other halogen atom) in this situation is that the electron withdrawing inductive effect is very strong, and is not overwhelmed by the electron releasing, resonance effect of the chlorine atom. Thus, the chloro substituent will have a relatively high charge density, identical in sign to that carried by the oxygen atom of the carbonyl moiety of the ester linking group. Thus, repulsion between these two will either cause a reduction in the coplanarity between the carbonyl moiety and the phenyl ring to which it attached, or twist the two phenyl rings out of plane by rotation about the phenol ether moiety carbon – oxygen bond. In either event, the nematic thermal stability of the **E–2Cl** molecule will be partially lost through the loss of conjugation and/or thickening of the molecule. 

Alternatively, if this repulsive situation is alleviated by a 2-chloro substituent lying on the opposite side of the molecule to the carbonyl function, the molecular breadth is markedly increased. Again, a reduction in the N – I transition temperature may be expected [11]. 

In the case of compound **E–3CH_3_** the intramolecular H – bonding between the 3– hydroxyl group and the azomethine nitrogen atom gives the molecules a rigid central core with the result that the aromatic rings adopt an almost coplanar orientation and the polarizability of the molecules is increased, so the effect of the hydroxyl group on the breadth of the molecule is lessened. This structure tends to favor the nematic phase to a greater degree than the other compound [12].

The opposite effect of the lateral group appears clearly when the hydrogen atom is replaced by a fluorine atom, as the ester compound **E–3F **shows smectic and nematic phases, like the unsubsitututed compounds, at a high temperature range. From all this we can conclude that the ratio of lateral to terminal attraction forces in this compound is high as compared to the substituted compounds **E–3CH_3_**, **E–3Cl**, **E–2Cl**, despite the opposite orientation of the dipole moments of the ester and fluoride groups and this can be explained by the fact that the fluoride atom does not increase the width of the molecule and that is due to the small size of this atom as compare to the other substituents. In addition, despite the fact that the C – F bond has a high dipole moment which acts through the long axis of the molecule, it cannot be cancelled totally by the dipole moment due to the ester group [13], so the resultant dipole moment along the longitudinal axis of the molecule approaches the resultant dipole moment through the lateral axis of the molecule, that is, the terminal attractive forces is almost the same as the lateral attraction forces, which make the two phases appear at low temperatures. The phase transition temperatures of all the compounds are summarized in Table 1. The enthalpy and entropy values for all transitions are presented in Table 2.

**Table 1:** The phase transitions of compounds (**°**C).

Compound	C→C	C→S_c_	S_c_→N	C→N	N→I	ΔT_S_	ΔT_N_
E-H	94.82	109.63	175.98	-	219.17	66.35	43.19
E-OH	-	-	-	103.23	217.41	-	114.18
E-3Cl	-	-	-	80.81	160.89	-	87.08
E-2Cl				77.92	160.00		80.77
E-CH_3_	-	-	-	89.13	165.84	-	76.71
E-F	-	80.3	154.44	-	203.33	74.14	48.89

S_c_ = Smectic N = Nematic; C = Crystal; ΔT_N _= Thermal range of nematic phase; ΔT_S _= Thermal range of smectic phase.

**Table 2:** The enthalpy and entropy values for the phase transitions of compounds.

Compound	C→C		C→S_C_		S_C_ → N		C → N		N → I	
	ΔH	ΔS	ΔH	ΔS	ΔH	ΔS	ΔH	ΔS	ΔH	ΔS
**E-H**	4.38	11.92	23.17	60.57	0.36	0.80	-	-	0.68	1.39
**E-OH**	-	-	-	-	-	-	32.95	87.58	0.54	1.07
**E-3Cl**	-	-	-	-	-	-	31.35	88.62	0.51	1.06
**E-2Cl**	-	-	-	-	-	-	30.24	86. 20	0.47	1.10
**E-CH_3_**	-	-	-	-	-	-	29.13	80.46	0.36	0.80
**E-F**	-	-	43.00	121.73	0.32	0.75	-	-	0.87	1.83

C = solid N =Nematic phase S_C_= Smectic phase I = Isotropic ΔH = KJ·mol^-1 ^ΔS=J·mol^-1^.K^-1^.

As seen in Figure 1, a nematic phase appears as a marble texture on heating and a Schlieren texture on cooling, while a smectic (Sc) phase appears as a Schlieren texture on heating and a Broken Fan Shaped texture on cooling (Figure 2).

**Figure 1:**
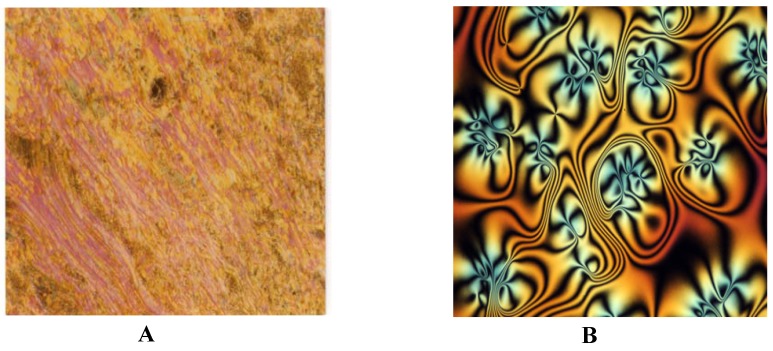
A. Marble texture for the nematic phase in heating at 115 °C for **E – OH; B.** Schlieren texture for Nematic phase in cooling at 203 °C for **E – OH**.

**Figure 2:**
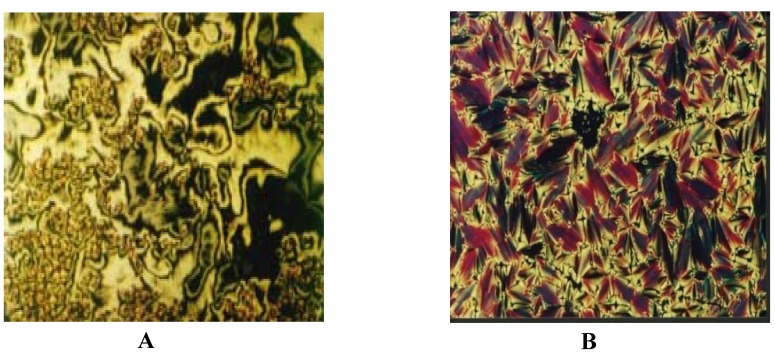
A. Schlieren texture for S_C_ phase in heating at 189 °C for **E –H; B.** Broken Fan Shaped texture for S_C_ phase in cooling at 209 °C for **E –H**.

## Experimental

### General

**Scheme 1:**
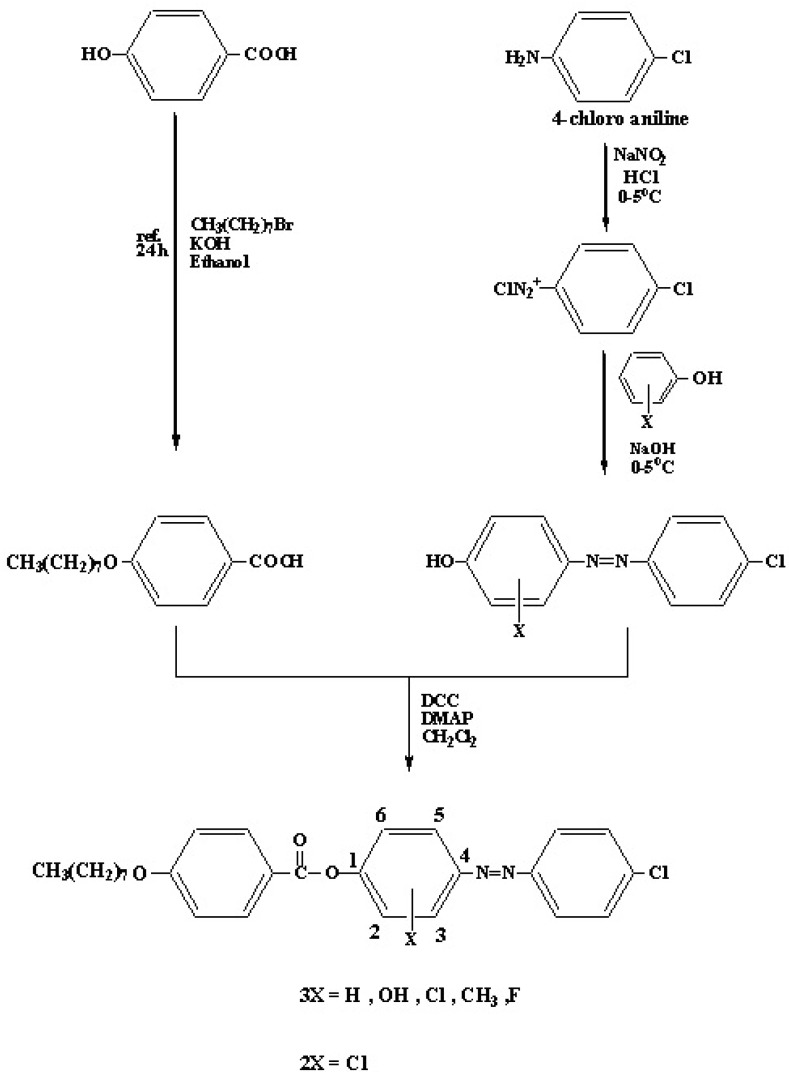
Synthesis of the compounds.

Infrared spectra were recorded as KBr pellets on a Buck – M500 spectrometer (Buck Scientific, USA). ^1^H-NMR and ^13^C – NMR spectra were recorded on a Gemini – 200 instrument using CDCl_3_ as solvent and TMS as internal standard (Varian, Germany). Elemental analysis was performed on a Euro Vector (Italy) EA 3000A instrument. The phase transitions were observed with a Leitz Laborlux 12 Pol optical microscope with polarized light in conjunction with a Leitz 350 hot stage (Germany) equipped with a Vario – Orthomat. Transition temperatures were determined using a Shimadzu 24 DSC–50 differential scanning calorimeter (Japan) with a heating rate of 10 °C min^-1^. 4‑ Octyloxybenzoic acid was synthesized by a modification of a literature method [14] (Scheme 1).

### Synthesis of azo compounds *A-3H, A-3OH, A-3CH_3_, A- 3F, A-3Cl* and *A-2Cl*

#### Diazotization of anilines

A solution of *p*-chloroaniline (10 mmol) and 3M HCl (8 mL) was heated gently, then water (10 mL) was added in order to dissolve the solid. The mixture was cooled to 0 °C in an ice bath with stirring. Some solid may reprecipitate, but the reaction will still work well if it is stirred. Freshly prepared 1M sodium nitrite solution (10 mL) was then added slowly with stirring. The rate of addition was adjusted so that the temperature of the solutions remains below 10 °C. The solution was kept in an ice bath and used immediately in the next step [15]. 

#### Coupling with phenols

A solution of one of the substituted phenols (10 mmol) in 1M NaOH (20 mL) was prepared and cooled in an ice bath. The diazonium salt from step 1 was then added slowly with stirring to the phenol solution. The reaction mixture was then left standing in the ice bath for at least 15 minutes until the crystallization is complete (giving a colored solid). The pH of the solution was adjusted with dilute HCl or NaOH solutions (0.1M) in order to induce precipitation. The orange azo dye was then collected and washed with cold water [15].

*4-[(4-Chlorophenyl)diazenyl]phenol* (**A–3H**): Yield 45%; m.p.: 154-156 ^o^C; IR (cm^-1^): 3,280 (OH), 1,600 (C=C); elemental analysis calc. for C_12_H_9_ON_2_Cl: %C 61.93, %H 3.87, %N 12.04; found: %C 61.63, %H 3.86, %N 11.94. 

*4-[( 4-Chlorophenyl)diazenyl]benzene-1,3-diol* (**A-3OH**): Yield 39%, m.p.: 195–197 °C; IR (cm^-1^): 3,260 (OH), 1,610 (C=C); elemental analysis calc. for C_12_H_9_O_2_N_2_Cl: %C 57.94, %H 3.62, %N 11.26; found: %C 57.74, %H 3.61, %N 11.24.

*4-[(4-Chlorophenyl)diazenyl]-3-methylphenol* (**A-3CH_3_**): Yield 32%; m.p.: 107–109 °C; IR (cm^-1^): 3,240 (OH), 1,600 (C=C); elemental analysis calc. for C_13_H_11_ON_2_Cl: %C 63.28, %H 4.46, %N 11.35; found: %C 62.98, %H 4.45, %N 11.32.

*4-[(4-Chlorophenyl)diazenyl]-3-fluorophenol* (**A-3F**): Yield 65%; m.p: 171–173 °C; IR (cm^-1^): 3,345 (OH), 1,600 (C=C); elemental analysis calc. for C_12_H_8_ON_2_ClF: %C 57.48, %H 3.19, %N 11.17; found: %C 57.28, %H 3.18, %N 11.16. 

*3-Chloro-4-[(4-chlorophenyl)diazenyl]phenol* (**A-3Cl**): Yield 53%; m.p.: 122–124 °C; IR (cm^-1^): 3,300 (OH), 1,600 (C=C); elemental analysis calc. for C_12_H_8_ON_2_Cl_2_: %C 53.93, %H 2.99, %N 10.48; found: %C 53.63, %H 2.95, %N 10.38. 

*2-Chloro-4-[(4-chlorophenyl)diazenyl]phenol* (**A-2Cl**): Yield 50%; m.p.: 162–164 °C; IR (cm^-1^): 3,480 (OH), 1,600 (C=C); elemental analysis calc. for C_12_H_8_ON_2_Cl_2_: %C 53.93, %H 2.99, %N 10.48; found: %C 53.53, %H 2.97, %N 10.47.

### Synthesis of esters

Solutions of 4-octyloxybenzoic acid (10 mmol), azo compound **A-3H**, **A-3OH**, **A-3CH_3_**, **A-3F**, **A-3Cl **or **A-2Cl **(10 mmol) and 1,3-dicyclohexylcarbodiimide (DCC, 55 mmol) in dry chloroform (50 mL) containing solid 4-dimethylaminopyridine (DMAP, 2.5 mmol) as catalyst were magnetically stirred at room temperature for 12 h. The byproduct (dicyclohexylurea) was filtered off under suction and the solvent was removed on a rotavapor. The crude product was recrystallized from hot ethanol [16]. 

*4-[(4-Chlorophenyl)diazenyl]phenyl-4-octyloxybenzoate* (**E-3H**): Orange solid; yield 43%; ^1^H-NMR δ: 0.76 (t, 3H, CH_3_), 1.18–1.69 [m, 12H, (CH_2_)_6_], 3.92 (t, 2H, OCH_2_), 6.83–8.04 (m, 4H, Ar-H); IR (cm^‑1^): 1,727 (C=O), 1,481–1,608 (C=C); elemental analysis calc. for C_27_H_29_O_3_N_2_Cl: %C 69.75, %H 6.24, %N 6.02. Found: %C 69.96, %H 6.32, %N 6.08.

*4-[(4-Chlorophenyl)diazenyl]-3-hydroxyphenyl-4-octyloxybenzoate *(**E-3OH**): Orange solid; yield 40%;^ 1^H-NMR δ: 0.90 (t, 3H, CH_3_), 1.32–1.82 [m, 12H, (CH_2_)_6_], 4.05 (t, 2H, OCH_2_), 6.95–8.15 (m, 11H, Ar-H), 12.98 (s, 1H, OH_ortho_); IR (cm^-1^): 1,732 (C=O), 1,481–1,604 (C=C); elemental analysis calc. for C_27_H_29_O_4_N_2_Cl: %C 67.42, %H 6.03, %N 5.82. Found: %C 67.89, %H 6.09, %N 5.92. 

*4-[(4-Chlorophenyl)diazenyl]-3-fluorophenyl-4-octyloxybenzoate *(**E-3F**): Orange solid; yield 62%; ^1^H-NMR δ: 0.89 (t, 3H, CH_3_), 1.28–1.82 [m, 12H, (CH_2_)_6_], 4.03 (t, 2H, OCH_2_), 6.90–8.30 (m, 11H, Ar-H); ^13^C–NMR: 14.164 (CH_3_), 22.710–31.851 (CH_2_)_6_, 68.454 (OCH_2_), 77.485 (Ar–C), 137.456 (C–-N), 151.137, 154.168 (C–O), 162.128 (C–Cl), 163.461 (C–F), 164.192 (C=O); IR (cm^-1^): 1,721 (C=O), 1,480–1,614 (C=C); elemental analysis calc. for C_27_H_28_O_3_N_2_ClF: %C 67.15, %H 5.80, %N 5.80. Found: %C 66.86, %H 5.77, %N 5.70. 

*4-[(4-Chlorophenyl)diazenyl]-3-methylphenyl-4-octyloxybenzoate* (**E-3CH_3_**): Orange solid; yield 40%; ^1^H-NMR δ: 0.89 (t, 3H, CH_3_), 1.31–1.83 [m, 12H, (CH_2_)_6_], 2.75 (s, 3H, CH_3_), 4.05 (t, 2H, OCH_2_), 6.95–8.17 (m, 11H, Ar-H); IR (cm^-1^): 1,732 (C=O), 1,481–1,602 (C=C); elemental analysis calc. for C_27_H_28_O_3_N_2_Cl_2_: %C 70.21, %H 6.47, %N 5.85. Found: %C 69.91, %H 6.46, %N 8.75.

*3-Chloro-4-[(4-chlorophenyl)diazenyl]phenyl-4-octyloxybenzoate* (**E-3C**l): Orange solid; yield 52%; ^1^H-NMR δ: 0.80 (t, 3H, CH_3_), 1.31–1.83 [m, 12H, (CH_2_)_6_], 4.05 (t, 2H, OCH_2_), 6.96–8.12 (m, 11H, Ar-H); IR cm^-1^: 1,721 (C=O), 1,480–1,614 (C=C); elemental analysis calc. for C_27_H_31_O_3_N_2_Cl: %C 64.92, %H 5.61, %N 5.61. Found: %C 64.72, %H 5.59, %N 5.50.

*2-Chloro-4-[(4-chlorophenyl)diazenyl]phenyl-4-octyloxybenzoate* (**E-2Cl**): Orange solid; yield 45%; ^1^H-NMR δ: 0.89 (t, 3H, CH_3_), 1.32–1.82 [m, 12H, (CH_2_)_6_], 4.06 (t, 2H, OCH_2_), 7.01–8.17 (m, 11H, Ar–H); IR cm^-1^: 1,748 (C=O), 1,481–1,604 (C=C); elemental analysis calc. for C_27_H_28_O_3_N_2_Cl_2_: %C 64.92, %H 5.61, %N 5.61. Found: %C 64.98, %H 5.62, %N 5.63.

## Conclusions

New azo mesogenic compounds with OH, CH_3_, Cl, and F side groups on the central benzene nucleus were synthesized. The study indicated that the type of lateral groups and their position adversely affects the mesomorphic properties of these compounds**.**
